# In Memory of Dr G. V. Black

**Published:** 1916-01

**Authors:** William P. Cooke, Henry H. Piper, Amos I. Hadley


### OBITUARY

IN MEMORY OF DR G. V. BLACK. It becomes the opportunity and joy of comparatively few members of a profession to know intimately its greatest leaders, but it is the privilege of all through imagination, sympathy and reverence to break down the barriers of time and space and enter into very vital relations with what is worthiest in the life of professional men.

With feelings like these the members of the American Academy of Dental Science desire at this time to honor the memory of Dr. Greene Vardiman Black whose recent death strikes from its honorary list a distinguished name and leaves the dental profession everywhere the poorer by his absence, but richer in the inheritance he has left behind him.

Dr. Black's life was unusually varied in its activities within the profession and singularly rich in its accomplishment. He was connected with three dental schools but his great work was done in connection with the Northwestern University Dental School at Chicago, in which he was a professor for twenty-four years and Dean from 1897 to the time of his death. Modest in demeanor, genial and kindly in his relation with his fellows he was yet filled with a passion to make real and practical what he felt to be important and to enrich the profession with the best of which he was capable. Honors and opportunity for service come unsought to such a man, and they came to him in full measure. He was president of the Illinois Dental Society, first president of the State Board of Dental Examiners, president of the National Dental Association and was awarded medals for his contribution to dental science and literature. His contributions to professional literature were especially numerous and important. For this and much besides it is a satisfaction to honor this eminent member of the dental profession, but it becomes a privilege to honor him in addition for his catholic spirit, his wide sympathy and his manliness. His life work retained interest for him to the last and with the memory of him there will always be mingled the thought of a well-rounded character.

William P. Cooke,

Henry H. Piper,

Amos I. Hadley.

